# Effect of a Large-Scale Production and Quality-Controlled Program for Texture-Modified Diets on Older Hospitalized Patients with Oropharyngeal Dysphagia

**DOI:** 10.3390/nu18040601

**Published:** 2026-02-11

**Authors:** Adrian Nuñez-Lara, Paula Viñas, Marta Cera, Marta Santiago, Laura Minguella, Abel Llovet, Pere Clavé

**Affiliations:** 1 Gastrointestinal Physiology Laboratory, Hospital de Mataró, Department of Medicine, Universitat Autònoma de Barcelona, 08304 Barcelona, Spain; anunezla@csdm.cat (A.N.-L.); pvinas@csdm.cat (P.V.); mcera@csdm.cat (M.C.); msantiago@csdm.cat (M.S.); lminguella@csdm.cat (L.M.); 2 Centro de Investigación Biomédica en Red de Enfermedades Hepáticas y Digestivas (Ciberehd), 08304 Barcelona, Spain; 3 SERHS FOODS SL, 08397 Mataró, Spain; abel.llovet@grupserhs.com

**Keywords:** dysphagia, texture modified diets, triple adaptation of the Mediterranean diet, pureed, soft and bite-sized, fork mashable, rheology, mPa·s

## Abstract

**Background/Objectives**: Several studies described how diets should be adapted to meet textural, nutritional and organoleptic needs of older people with oropharyngeal dysphagia. However, few studies have evaluated the implementation of texture-modified diets (TMD) in a real clinical context. In 2024, a TMD program was introduced in a 400-bed hospital. The aim of this study was to describe the impact in production, texture standardization and acceptance of this program. **Methods**: This is an observational study that compares the TMD data of 2023 versus 2024. In this period, AI techniques and clinical staff training were implemented to increase TMD prescriptions. A quantitative weekly quality control was carried out to standardize the rheological and textural properties of the TMD. Qualitative questionnaires were used to evaluate acceptance and palatability in both years. **Results**: The number of TMD meal services served increased in 51.60% (9766 in 2023, 14,806 in 2024). Viscosity range variability in thick purees (IDDSI Level 4, target shear viscosity of 1500 ± 20% mPa·s) was reduced from 600–4800 mPa·s in 2023 (58.74% variability) to 1000–2400 mPa·s in 2024 (27.91% variability). Fork-mashable TMD (IDDSI Level 6) presented limitations in standardization, due to the lack of quantitative reference values for textural parameters. The meal intake consumption remained around 70% in both years. **Conclusions**: The large-scale hospital TMD production program was associated with an increased number of patients with dysphagia receiving TMD, reduced texture variability, and high levels of palatability and patient acceptability. These process-level improvements are expected to support swallowing safety, although clinical outcomes were not directly assessed.

## 1. Introduction

Oropharyngeal Dysphagia (OD) is a swallowing disorder characterized by impaired bolus transport from the mouth to the esophagus, potentially leading to aspiration or choking, and is classified as a digestive disorder by the World Health Organization [1]. OD can affect up to 55% of hospitalized older patients and is frequently underdiagnosed and undertreated [1,2,3,4,5]. Complications of OD related to safety impairments include recurrent and/or severe respiratory infections and, in many cases, increased mortality. Efficacy impairments can lead to malnutrition and dehydration, along with longer hospital stays, readmissions, and higher mortality rates in older adults [6,7].

The compensatory treatment for OD involves increasing fluid viscosity through thickening products (TP) and texture-modified diets (TMD) [8]. Early detection using artificial intelligence (AI) algorithms [9] or screening tests such as the Volume-Viscosity swallowing test (V-VST) [10,11] should be followed by standardized multidisciplinary protocols to confirm OD and adjust fluid viscosity and diet texture, with the aim of supporting swallowing safety and adequate nutritional intake. Recent studies showed that hospitalized older patients with OD often have severe swallowing safety impairments, frailty, reduced functional capacity, multiple comorbidities, malnutrition, dehydration, and poor oral hygiene [12]. The primary goal of compensatory treatment is to reduce and/or minimize the most common complications of OD while addressing patients’ therapeutic needs [13,14], ensuring adequate intake of calories, proteins, and nutrients through TMD and adapted fluid viscosities as part of a comprehensive care approach [13]. However, the effectiveness of compensatory strategies depends not only on prescription but also on the quality, consistency, and acceptability of the diets provided.

TMD are a fundamental tool for support swallowing safety in patients with chewing and/or swallowing disorders. The primary issues stem from TMD being visually unappealing and difficult to identify. In general, there is some resistance to these diets, leading to poor compliance with dietary prescriptions [15]. Additionally, blending foods can result in nutrient loss and altered taste, along with a higher risk of foodborne illness due to excessive handling. Certain foods, particularly protein-rich ones, are difficult to blend, leading to repetitive meal options. These factors contribute to low adherence and an increased risk of malnutrition in OD patients [16]. To address these limitations, Costa A. et al. [17] proposed a nutritional model based on the triple adaptation of the diet, integrating: (1) rheological adaptation (texture and viscosity); (2) nutritional adaptation (water, calories, and proteins); and (3) organoleptic adaptation to enhance adherence. A total of 296 recipes (16 weekly menus) were developed, demonstrating that traditional Mediterranean cuisine can be adjusted to meet OD patients’ rheological, nutritional, and hydration needs, considering their chewing ability, swallowing function, and nutritional status. This adaptation was also reproducible at home.

Different dietetic institutions around the world have proposed ways of classifying TMD based on their descriptive characteristics, such as the British Dietetic Association (BDA) [18], the Japanese Dysphagia Diet (JDD) [19] or the National Dysphagia Diet (NDD) [20]. In particular, the BDA divided them into four groups: Texture B (liquid puree), Texture C (thick puree), Texture D (chopped and moist) and Texture E (fork-mashable) [18]. These groups present a gradient of liquid to solid textures, but Textures C and E are mandatory nomenclatures in any OD patient care facility [21]. Texture C is a thick and dense puree, mainly intended for people with swallowing problems. Texture E is a very soft solid food that requires low chewing, mainly intended for people with difficulties in the masticatory process and bolus preparation [18].

Recently, many national scales, such as the BDA or the NDD, have standardized their criteria and have adopted the International Dysphagia Diet Standardisation Initiative (IDDSI) classification [22], which proposes classifying food products into levels from 0 (liquid) to 7 (solid) [23]. The College of Speech Therapists and the College of Nutritionists in the UK have defined BDA levels B, C, D and E as equivalent to IDDSI levels 3, 4, 5 and 6, respectively [22].

However, as these classifications are based on subjective criteria, they are imprecise and almost impossible to reproduce in industrial kitchens for quality control of food texture. Two products with the same parameters can be classified at different texture levels or, on the contrary, TMD with a very wide range of parameters can be found within the same texture level [24]. This limitation poses a significant challenge for large-scale hospital food production. Quantitative characterization of the rheological and textural properties of the diet using the International System of Units (SI) for viscosity and texture results in a much more precise analysis, capable of making an objective assessment of the swallowing safety of a TMD.

It have been demonstrated that shear viscosity is the main factor linked with the therapeutic effect of alimentary fluids, as thickened fluids or texture-modified purees (Texture C) [25]. The optimal viscosity level for purees was identified at 1500 mPa·s ± 20%, a range that has been associated with high levels of swallowing safety in previous clinical studies [26,27].

The most important textural properties are maximum force, adhesiveness and cohesiveness [17]. They have a relevant role during the swallowing of fluid TMDs (Texture C), but they are the main parameters involved in the swallowing security of solid TMDs (Texture E). However, this parameters are not as well studied as viscosity, and the optimal levels to guarantee safe swallowing are not known yet. Studies are currently being carried out on patients with OD to better understand the safety parameters of solid TMD [28].

All of this recent research is contributing to our understanding of how adapted diets should be designed to ensure maximum safety and efficacy for patients, but this knowledge is meaningless if it does not translate into improved clinical practice. Generally, adapted diets in care centers do not receive the attention they require. Few studies have evaluated the outcomes of an implemented protocol for improving the adapted diets served to their patients.

At the Mataró University Hospital, efforts have been made to provide high-quality TMD based on the triple adaptation of the Mediterranean diet. In 2023, however, a TMD improvement protocol was introduced. The combination of AI screening tool for OD (AIMS-OD) [9] and clinical diagnosis with V-VST [10,11] has been routinely implemented to increase OD detection and, consequently, texture adaptation prescriptions. A large-scale production and quality controlled program for the TMD was implemented, initially in collaboration with Arcasa, S.L catering until December 2023 and then with SERHS Food S.L. This process included the design, adaptation and incorporation of new and improved seasonal full TMD menus, a systematic quality-control program of the rheological and textural parameters of the TMD and patients’ acceptability evaluation.

Given that few studies have been published that include both qualitative and quantitative evaluations of TMD and their impact on the OD population, the main aim of the present study was to describe and evaluate the impact of the large-scale production and standardization program on the hospital’s TMD by comparing the status of the diets in 2023 (before protocol implementation) and 2024 (after protocol implementation). The study assesses whether TMD are being served to more people who need them and whether their quality has improved, which includes a standardization of rheological properties around optimal values and a positive impact on palatability and acceptability by patients with OD.

## 2. Materials and Methods

This work is an observational, descriptive, multimethod evaluation of the implementation of a hospital-wide TMD program. It comprises three complementary components designed to provide a comprehensive view of the process. First, a retrospective observational analysis of TMD prescriptions in 2023 and 2024 was performed to describe the patterns of use. Second, a quantitative characterization of the rheological and textural properties of TMDs was conducted through in vitro observational testing to document product consistency and quality. Finally, a qualitative study using patient questionnaires assessed the organoleptic, palatability and acceptance of TMD. A detailed disclosure of conflicts of interest is provided in the Conflict of Interest statement (page 19).

### 2.1. Prescriptions

To evaluate and monitor the large-scale production of TMD, a monthly record was kept of the menus produced and delivered, categorized by the type of diet prescribed. This record covered the years 2023 and 2024, allowing a comparative analysis between both years. Each month, the number of meals served (which included breakfast, lunch, afternoon tea and dinner during the same day for each patient) and the specific type of diet were documented. In addition, a systematic dysphagia screening method based on artificial intelligence (AIMS-OD) [9] was implemented, along with increased use of clinical assessment through the V-VST [10]. The diagnostic performance of the AI-based tool (sensitivity, specificity, AUC) were not evaluated during this study period. These measures were expected to enhance the identification of patients with OD and consequently increase the appropriate prescription of TMD.

Statistical differences between the number of TMD prescribed in 2023 versus those prescribed in 2024 were assessed using a parametric *t*-test. Significant differences were considered at *p* < 0.05. All statistical analyses were performed using GraphPad Prism 6.0 (GraphPad Software, San Diego, CA, USA).

### 2.2. Texture-Modified Diets

A one-week menu rotation (Monday to Sunday) was implemented. Two seasonal menus were available: summer and winter. The summer menu included 28 main dishes (first and second courses served at lunch and dinner), while an additional 16 dishes were rotated during the winter season. Four texture-modified breakfasts were also included in rotation. On average, the daily nutritional contribution of the meals was 1700–1800 kcal and 70–90 g of protein.

### 2.3. Quantitative Studies

#### 2.3.1. Study Design

The quantitative data were obtained from a quality control that was performed weekly on the TMD. It was a single-center, in vitro observational study. The rheological properties of the TMD in Texture C were analyzed in a rheometer, and shear viscosities at 50 s^−1^ (shear rate of oral cavity [29]) and 300 s^−1^ (shear rate of laryngeal vestibule [29]) were obtained to assess the oral and pharyngeal phase, respectively. The textural properties of the TMD in Texture C and E were analyzed in a texturometer, and three parameters were obtained: maximum force (N), adhesiveness (N·s) and cohesiveness.

#### 2.3.2. Materials

Twenty components of the hospital’s TMDs cooked separately in the kitchen were analyzed (specific ingredients are presented in Appendix A, Table A1). The TMDs were provided by Catering Arcasa, S.L (2023) and SERHS Food S.A. (2024). All batches of the same TMD followed a recipe with pre-established quantities, varying only the amount of TP and water to modify viscosity and texture without affecting the nutritional components. Two textural levels were selected to prepare each TMD, according to the criteria used in our previous studies [17,26,27,28]:

▪ Texture C: Thick purée (Level IDDSI 4)
▪ Texture E: Fork-mashable (Level IDDSI 6)

#### 2.3.3. Equipment

Shear viscosity. A rotational rheometer (RheolabQC) with temperature control (C-PTD 180/AIR/QC) and software Anton Paar RheoCompass (version 1.30. Ink) was used to assess the shear viscosity of the TMD in Texture C. The rotor sensory system CC17/QC was selected to carry out the analysis.

Textural parameters. A texturometer (TA.XT plus C 650) was used to assess the textural properties of the alimentary fluids. The probe selected for the analysis was an aluminum cylinder probe 35 mm in diameter, combined with a plastic cup of 50 mm internal diameter where the food was placed.

#### 2.3.4. Methodology

The TMDs were transported in circular bowls inside a hermetic plate from the hospital’s kitchen to the rheology laboratory. The temperature was assessed with a thermometer and, if it was 40–50 °C, the test was started.

All the tests were performed by triplicate and the mean and the standard deviation (SD) were calculated. All results are presented as mean ± SD.

Shear viscosity measurement. This test was only performed for Texture C of the TMDs. 5 mL of the analyzing pureé C were introduced into the gap of the rotor sensory system and connected to the rheometer. The temperature control was set at 40 °C, excepting product I, which was a cold TMD and its temperature was set at 25 °C. The test started and the shear viscosity was measured for a shear rate frame ranging from 0.1 to 1000 s^−1^. Viscosities at 50 s^−1^ were interpolated from the regression line, according to our previous studies.

**Figure 1:**
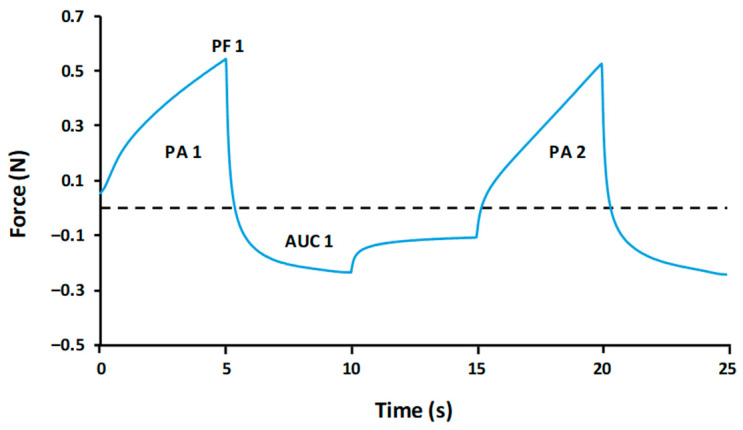
Texture Profile Analysis (TPA) graph. Peak force 1 (PF 1) indicates maximum force (N), Area under the curve 1 (AUC 1) indicates adhesiveness (N·s) and the division of positive area 2 (PA 2) by 1 (PA 1) indicates cohesiveness.

Textural characterization. This test was performed on both viscosity levels (C and E) of the TMDs. In total, 30 g of the analyzing product was placed in the plastic cup of the texturometer. A texture profile analysis (TPA) was performed. TPA test consists of a double compression simulating two bites, giving results of force vs. time (Texture Expert for Windows v. 1.05 software, Stable Micro Systems, Godalming, UK). The textural properties were extrapolated from the graph (Figure 1) obtained as follows:

▪ Maximum force (N): Peak force 1
▪ Adhesiveness (N·s): Area under the curve 1
▪ Cohesiveness (adimensional): Positive area 2/Positive area 1

#### 2.3.5. Quality Control Data Summary and Analysis

To summarize the results of the quality control, a retrospective analysis of all data collected in 2023 and 2024 was performed. The annual results of each dish were assessed, calculating the mean and the SD of all weekly analysis, for each rheological and textural parameter. The mean of all TMD results were assessed, as well as the lowest and highest value for each parameter.

The variability of rheological and textural parameters between the TMD of each year was evaluated using the Coefficient of Variation. No inferential statistics were applied to rheological or textural parameters because each weekly measurement corresponds to a different batch and is not a repeated measure of the same product. Therefore, the data reflect population-level dispersion and not sample variance. Batch-level variability between years was assessed using all weekly measurements pooled by year. An exploratory test for equality of variances was applied to compare dispersion between 2023 and 2024. However, given the non-experimental nature of the data and potential deviations from normality, results were interpreted with caution and primarily supported by descriptive variability metrics.

### 2.4. Qualitative Studies

Several qualitative evaluations were performed for both Texture E and C during 2023 and 2024. In 2023, simple questionnaires were given, including questions about temperature, taste, appearance and texture (rated from 0 to 5), as well as % of intake of the menu. However, in 2024, *Face Likert Scales* were used, a more precise and validated questionnaire. It also consists of a questionnaire that rates items such as texture, taste, appearance of each dish, as well as the oral residue and mastication sensation from 1 to 5 (Supplementary Material, Figure S1). Along with both questionnaires, the percentage of intake for each menu was also recorded. Due to the use of different survey tools across years, no formal statistical comparison was attempted.

The questionnaires were administered by dietitians and were given to older hospitalized patients with OD who required a TMD in Texture E or C and who were able to answer the questions independently and/or with minimal support from a caregiver/family member admitted to the geriatric unit. There were no further inclusion/exclusion criteria in order to select the entire population that ingested the TMDs, and not select a specific profile of patients that could generate a bias. The surveys were conducted orally and written down during meals, as it attempted to assess the appearance and sensory characteristics of the dishes from the moment they were served. No identifiable demographic or clinical data were collected as part of the questionnaire, which precluded a detailed characterization of the respondents.

## 3. Results

### 3.1. Prescriptions

**Figure 2:**
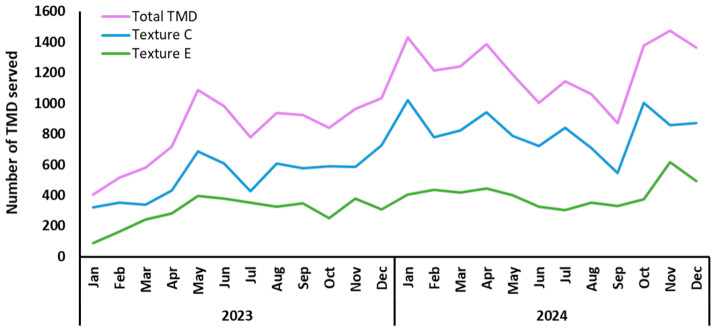
Texture-modified diets meals served per month during 2023 and 2024, presented by textures and as total prescriptions. TMD: texture-modified diets; Jan: January; Feb: February; Mar: March; Apr: April; Jun: June; Jul: July; Aug: August; Sep: September; Oct: October; Nov: November; Dec: December.

The number of TMD prescriptions gradually increased during 2023, as the model was expanded and integrated into various hospital units. By the end of the year, the number of TMD served reached a total of 9766 meals (6255 Texture C and 3511 Texture E) (Table 1), with an average of 26.76 ± 7.15 servings per day (Figure 2). As the model was consolidated in 2024, the total number of TMD meals served was 14,806 (9916 in Texture C and 4890 in Texture E) (Table 2), with an average of 40.45 ± 6.08 servings per day (Figure 2), a significant increase of 51.60% (*p* < 0.0001).

### 3.2. Quantitative Studies

#### 3.2.1. Year 2023

##### Texture C

**Figure 3:**
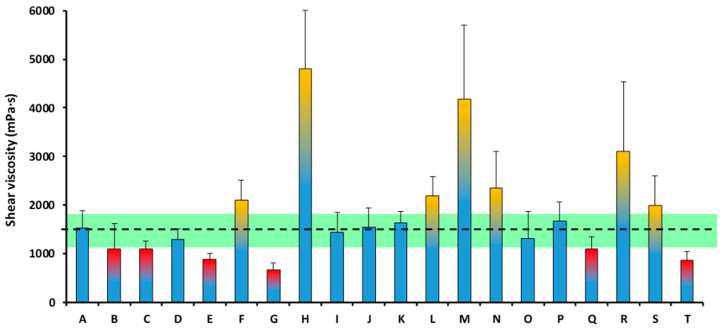
Shear viscosity of each TMD of Texture C at 50 s^−1^ in the year 2023. The viscosity range of 1500 ± 20% mPa·s (1200–1800 mPa·s), previously described as optimal, is marked with a discontinuous line and a green stripe. The columns tipped in yellow presented a shear viscosity over 1800 mPa·s. The columns tipped in red presented a shear viscosity below 1200 mPa·s. Values outside the target range indicate deviation from predefined viscosity target and do not necessarily represent clinical safety thresholds.

The mean shear viscosity of all the analyzed TMDs was 1842.12 ± 1082.11 mPa·s, which is outside the 1500 ± 20% mPa·s viscosity range, previously described as optimal in relation to swallowing safety (1200–1800 mPa·s). The shear viscosity of the TMDs ranged between 670.43 mPa·s (Product G) and 4809.85 mPa·s (Product H) (Figure 3).

The results of the shear viscosity and the textural parameters are presented in Supplementary Material, Table S1. The mean maximum force between the TMD was 0.59 ± 0.19 N. All the products revealed maximum force values between 0.37 N (Product G) and 1.20 N (Product R). The mean adhesiveness among all the Texture C products was 0.81 ± 0.20 N·s, ranging between 0.35 N·s (Product Q) and 1.26 N·s (Product M). The mean cohesiveness of all TMD was 0.77 ± 0.04, and ranged between 0.66 (Product R) and 0.82 (Product C).

##### Texture E

**Figure 4:**
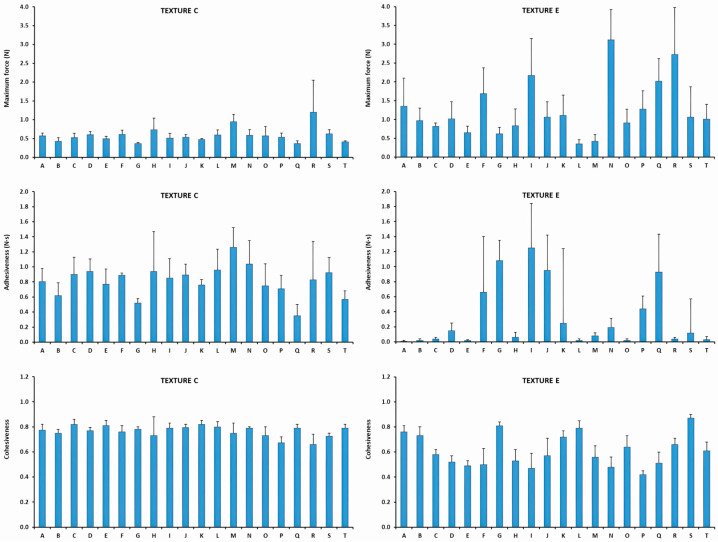
Textural characterization of each TMD at Texture C and E. Maximum force values are presented above, adhesiveness values between and cohesiveness below (year 2023).

The textural parameters results are graphically presented in Figure 4. Numerical textural values can be found in Supplementary Material, Table S2. The mean maximum force between the TMD was 1.26 ± 0.74 N, ranging between 0.35 N (Product L) and 3.12 N (Product N). The mean adhesiveness among Texture E products was 0.32 ± 0.41 N·s, with a range between 0.01 N·s (Product A) and 1.25 N·s (Product I). The mean cohesiveness value of Texture E TMD was 0.61 ± 0.13 ranging between 0.42 (Product P) and 0.87 (Product S).

#### 3.2.2. Year 2024

##### Texture C

**Figure 5:**
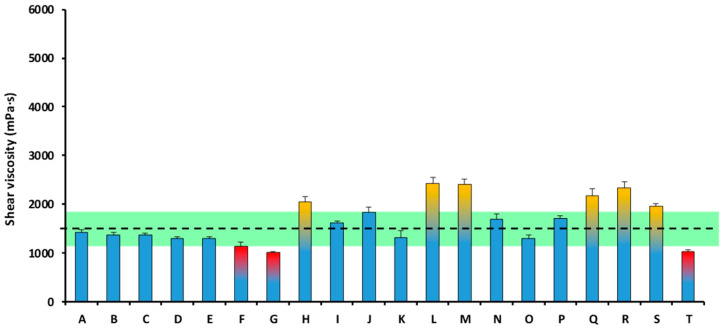
Shear viscosity of each TMD of Texture C at 50 s^−1^ in the year 2024. The viscosity range of 1500 ± 20% mPa·s (1200–1800 mPa·s), previously described as optimal, is marked with a discontinuous line and a green stripe. The columns tipped in yellow presented a shear viscosity over 1800 mPa·s. The columns tipped in red presented a shear viscosity below 1200 mPa·s. Values outside the target range indicate deviation from predefined viscosity target and do not necessarily represent clinical safety thresholds.

The mean shear viscosity of all the analyzed TMDs was 1634.71 ± 456.25 mPa·s, which is inside the 1500 ± 20% mPa·s viscosity range, previously described as optimal in relation to swallowing safety (1200–1800 mPa·s). The shear viscosity of the TMDs ranged between 1006.36 mPa·s of (Product G) and 2415.91 mPa·s (Product L) (Figure 5).

The results of the shear viscosity and textural parameters are presented in Supplementary Material, Table S3. The mean maximum force between the TMD was 0.53 ± 0.11 N. All the products revealed maximum force values between 0.40 N (Product G) and 0.88 N (Product R). The mean adhesiveness among the Texture C products was 0.78 ± 0.13 N·s, ranging between 0.53 N·s (Product F) and 1.02 N·s (Product P). The mean cohesiveness value of the TMDs was 0.78 ± 0.04. All the TMDs ranged between 0.70 (Product P) and 0.84 (Products L and M).

##### Texture E

**Figure 6:**
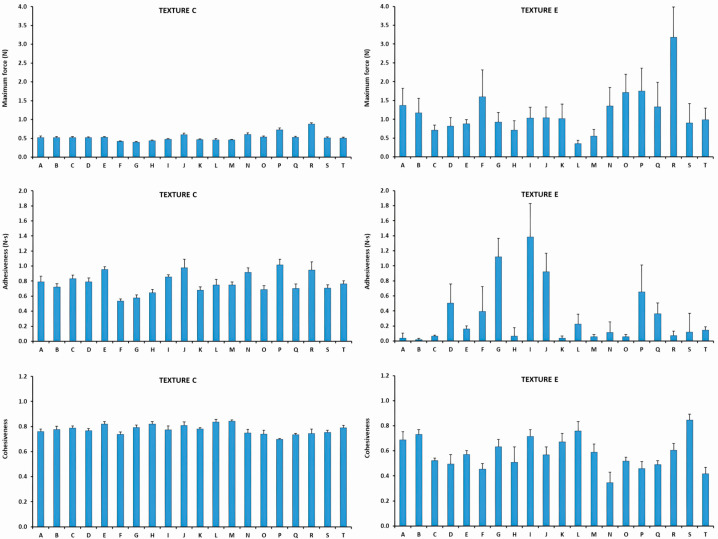
Textural characterization of each TMD in Texture C and E. Maximum force values are presented above, adhesiveness values between and cohesiveness below (year 2024).

The results of the textural parameters are graphically presented in Figure 6. Numerical textural values can be found in Supplementary Material, Table S4. The mean maximum force between the TMD was 1.17 ± 0.60 N. All the products revealed maximum force values between 0.36 N (Product L) and 3.18 N (Product R). The mean adhesiveness among the Texture C products was 0.33 ± 0.40 N·s, with a range between 0.02 N·s (Product B) and 1.39 N·s (Product I). The mean cohesiveness value of the TMD was 0.58 ± 0.13 ranging between 0.35 (Product N) and 0.85 (Product S).

#### 3.2.3. Year Comparison

The following results describe year-to-year changes in rheological and textural variability as indicators of production consistency and quality control, rather than as measures of clinical efficacy or patient-level outcomes. The variance comparisons and their associated *p*-values are exploratory and should not be interpreted as evidence of causal improvement.

##### Texture C

**Figure 7:**
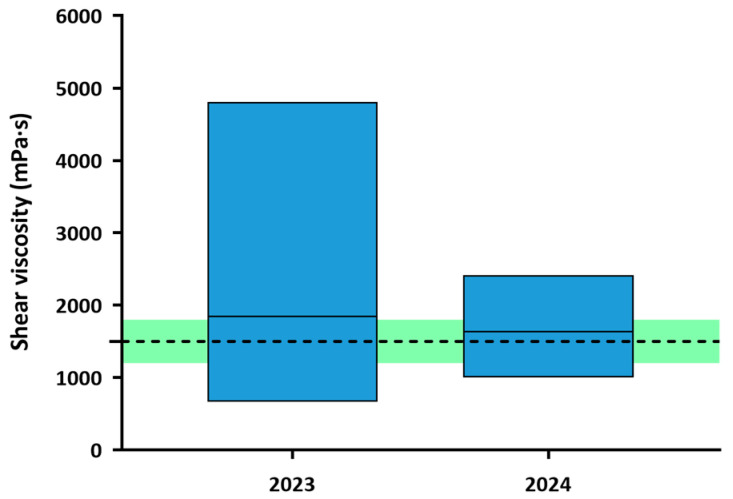
Shear viscosity range of TMD for each year at Texture C. The columns represent the range in which some viscosities were measured, marking the lower limit at the lowest value and the upper limit at the highest. The center line represents the average of the viscosity of all TMD. The viscosity range of 1500 ± 20% mPa·s (1200–1800 mPa·s), previously described as optimal, is marked with a discontinuous line and a green stripe. Values outside the target range indicate deviation from predefined viscosity target and do not necessarily represent clinical safety thresholds.

Figure 7 illustrates that shear viscosity range was reduced from 670.43–4809.85 mPa·s in 2023 to 1006.36–2415.91 mPa·s in 2024. Specifically, the annual coefficient of variation decreased significantly (*p* = 0.0004) from 58.74 to 27.91%, indicating greater consistency across production batches and TMD. Furthermore, the mean viscosity among all Texture C TMD was 1842.12 ± 1082.11 mPa·s in 2023, a value outside the proposed optimal parameter (1200–1800 mPa·s), while in 2024, the mean was 1634.71 ± 456.25 mPa·s, a value within the optimal range.

**Figure 8:**
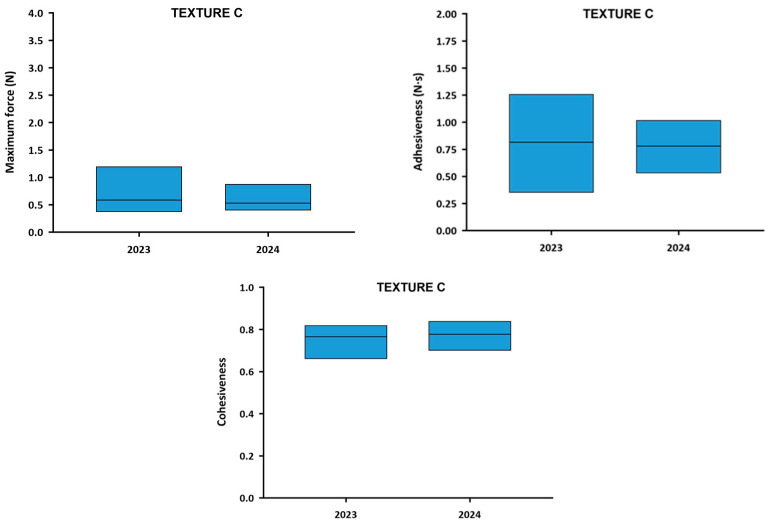
Ranges of textural parameters of Texture C TMD for each year. The columns represent the range in which some parameters were measured, marking the lower limit at the lowest value and the upper limit at the highest. The center line represents the average of the textural parameters among all TMD.

The comparison of the textural parameters of Texture C TMD between 2023 and 2024 is presented in Figure 8. Although the average maximum force barely varied between the 2 years (0.59 N in 2023 and 0.53 N in 2024), the range of values was significantly reduced (*p* = 0.0153) in 2024 (from 33.18% to 20.54%). This pattern also occurred in adhesiveness, which showed similar mean value (0.81 N·s in 2023 and 0.78 N·s in 2024) and less variability between samples in 2024 (from 24.48% to 17.07%), but it was not significant (*p* = 0.0861). Cohesiveness mean value remained very similar in both years (0.77 in 2023 and 0.78 in 2024), and presented a slight non-significant reduction (*p* = 0.4681) in the variability of results (from 5.78% to 4.81%). All these differences in rheological and textural parameters should be interpreted as descriptive indicators of changes in production variability between years and not as evidence of causal improvement or clinical benefit.

##### Texture E

**Figure 9:**
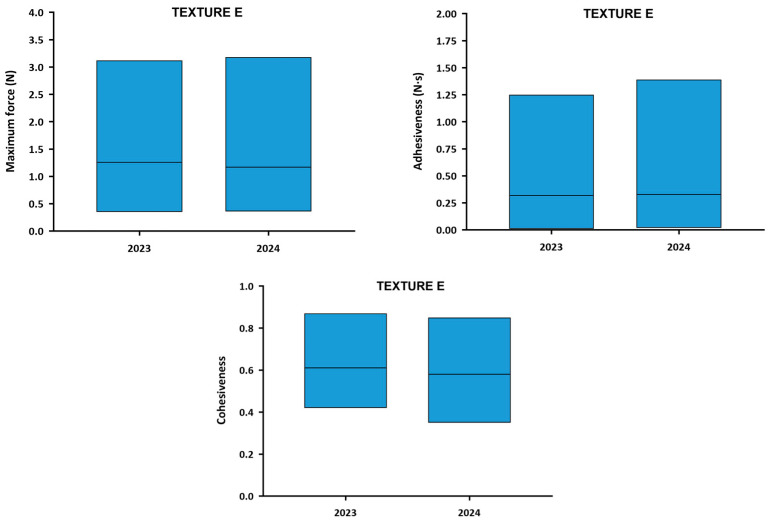
Ranges of textural parameters of TMD at Texture E each year. The columns represent the range in which some parameters were measured, marking the lower limit at the lowest value and the upper limit at the highest. The center line represents the average of the textural parameters among all TMD.

Figure 9 represents the comparison of the textural parameters of Texture E TMD between 2023 and 2024. The average maximum force was very similar in both years (1.26 N in 2023 and 1.17 N in 2024) and presented a slight non-significant reduction (*p* = 0.3780) in the variability of results (from 58.58% to 51.40%). This pattern also occurred in adhesiveness, which showed similar values of both mean value (0.32 N·s in 2023 and 0.33 N·s in 2024) and variability between years (from 130.14% to 122.06%) (*p* = 0.8688) and cohesiveness, mean value (0.61 in 2023 and 0.58 in 2024) and variability of results (from 21.27% to 21.95%) (*p* = 0.9230). This lack of standardization between both years does not imply negative clinical outcomes, but rather evidence that the consistency of the texture has not been increased between production batches.

### 3.3. Qualitative Studies

#### 3.3.1. Evaluation During 2023

**Figure 10:**
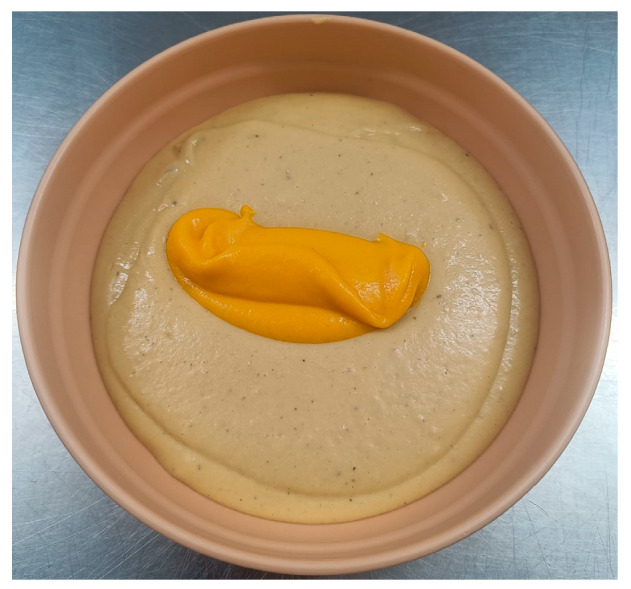
Stewed Turkey in Texture C.

**Figure 11:**
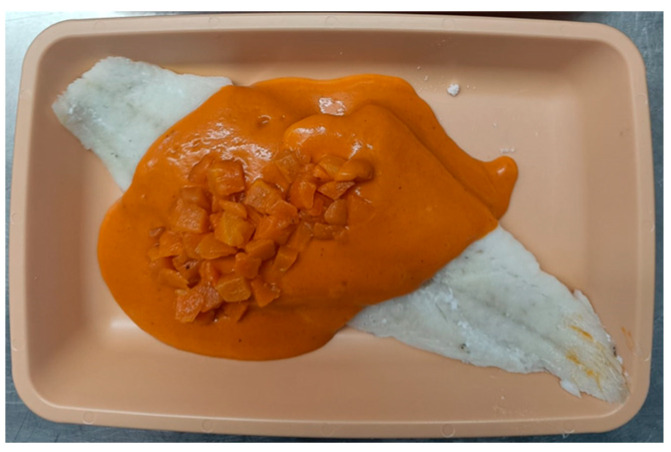
Hake with vegetable ratatouille in Texture E.

In 2023, 257 questionnaires were conducted, the average score of which was 4.3 ± 1 out of 5, while the texture opinion reached an average of 4.8 ± 0.65 out of 5. The average percentage of meal intake consumption was 70%, suggesting good acceptance of the diets in terms of the volume consumed by the patients. Among the best rated dishes served were Stewed Turkey in Texture C (Figure 10) and Hake with vegetable ratatouille in Texture E (Figure 11), with scores of 4.75 ± 0.5 and 4.4 ± 0.65 out of 5, respectively.

**Figure 12:**
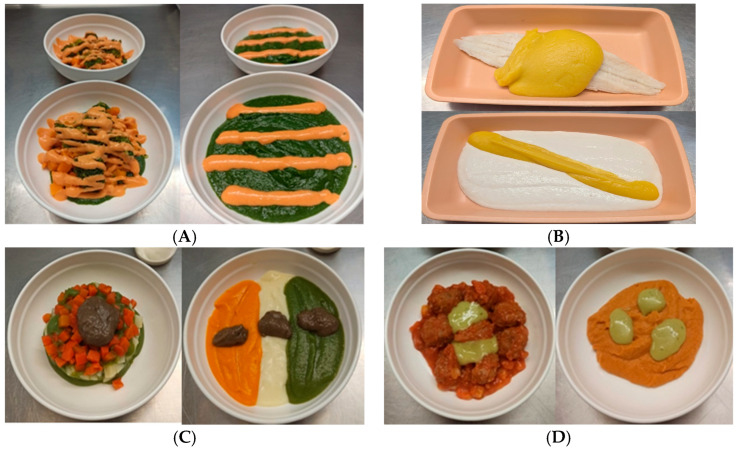
Examples of texture-modified diets served at the Mataró Hospital. Texture E (**left**) and Texture C (**right**). (**A**) Vegetables with romesco sauce. (**B**) Hake with lemon sauce. (**C**) Vegetables with mushroom sauce. (**D**) Garden-style meatballs.

The total caloric value of the daily diet was 1899.24 kcal, with a protein intake of 77.73 g per day. These values were carefully adjusted to meet the nutritional needs of patients with OD, ensuring that the menus not only met their chewing and swallowing capabilities but also fulfilled their energy and protein requirements. The presentation of the dishes was also thoroughly reviewed. Figure 12 shows the appearance of some example dishes.

#### 3.3.2. Evaluation During 2024

Regarding the qualitative evaluation conducted in 2024, 72 surveys based on Face Likert Scale were administered to older patients with OD admitted to the Mataró Hospital who were receiving TMD. The results of this evaluation revealed an average score of 3.6 ± 0.7 out of 5 for overall patient satisfaction with the TMD, with better ratings for dishes with Texture E (3.8 ± 0.5 out of 5) compared to those with Texture C (3.5 ± 0.8). In the organoleptic section of the surveys, which included attributes such as taste, texture, and appearance, the scores were also high (3.6 ± 0.5 out of 5), indicating positive acceptance of the sensory characteristics of the menus. Additionally, the scores for ease of chewing and swallowing and perception of oral residue during diet intake reached an average of 3.8 ± 0.6 out of 5. The recorded intake percentage in this second evaluation was 66.2%. These results should be interpreted with caution, given the limited sample size and response rate of the 2024 qualitative survey.

**Figure 13:**
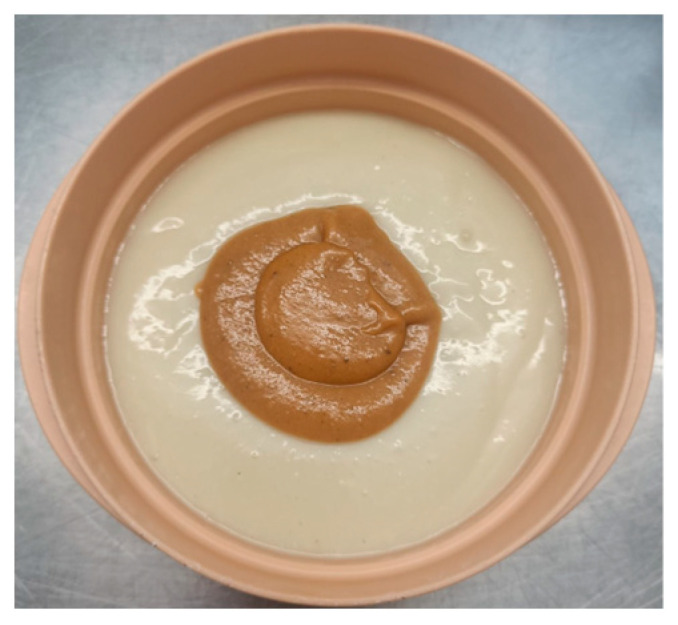
Macaroni Bolognese.

**Figure 14:**
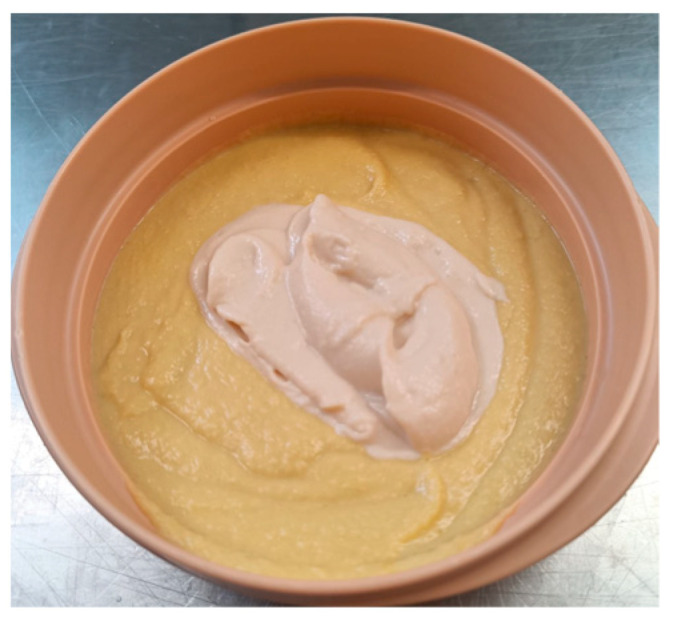
Chickpeas with tuna and hard-boiled eggs.

The dish that received the highest score in Texture E was Stewed red lentils, followed by Panga with samfaina (vegetable stew); in Texture C, both Macaroni Bolognese (Figure 13) and Chickpeas with tuna and hard-boiled eggs (Figure 14) received the same score. However, patients did not rate the Fideuá (a traditional Spanish noodle dish) in Texture E as well, nor the Potato nests with vegetables in Texture C.

## 4. Discussion

OD is a commonly underdiagnosed and undertreated condition in hospitalized patients, especially among the geriatric population. This syndrome poses a significant health risk, as it can lead to severe complications such as recurrent respiratory infections, malnutrition, dehydration, and prolonged hospital stays. TMD are widely used in the management of OD and have been described as a strategy to support swallowing safety and reduce aspiration risk. However, they are also associated with potential nutrient inadequacies and are frequently rejected by patients due to limited sensory appeal. This study focused on standardizing the properties of TMD served in a general hospital, carrying out quality controls to quantify the rheological and textural parameters of the TMD present in the hospital menu, and evaluating the acceptance and adherence of the TMD through qualitative questionnaires.

Between 2023 and 2024, the number of TMD meals served increased by more than 50%. This change occurred alongside the implementation of several organizational measures aimed at improving the identification and management of OD. During this period, the hospital introduced more systematic screening protocols, including the use of AI-based digital decision-support tools integrated into the electronic health record [9], which facilitated the identification of patients at potential risk. In parallel, targeted training sessions were provided to healthcare staff to promote the consistent use of validated bedside screening tools, such as the V-VST [10]. Together, these measures supported a more structured and standardized approach to OD screening, which may have contributed to earlier recognition of swallowing difficulties and a more appropriate allocation of TMD meals.

Second, this study showed that large-scale production in TMDs was possible without significant modifications in infrastructure or staffing, although some additional assistance for the cook was required. All TMD continued to be prepared in the hospital kitchen by a professional chef specialized in this type of diets, following standardized procedures that remained unchanged throughout the study period. The rise in TMD distribution reflects the higher number of prescriptions resulting from improved OD detection which the kitchen production mechanisms managed to meet. This finding may suggest that hospital kitchens are generally capable of scaling up TMD production when needed, and that one of the main limiting factors in providing appropriate diets is the underdiagnosis of dysphagia.

The observed changes in the rheological and textural properties of the TMD served in 2023 and 2024 suggests that the systematic monitoring and control of texture can increase inter-batch consistency without reducing acceptability and palatability. The results not only reveal a considerable reduction in the variation among textural properties that the patients received throughout the menu, but also adjusted their range to the values that previous studies have associated with improved swallowing safety. These positive results may be explained by two major factors. First, finding an objective, numerical and quantitative optimal shear viscosity that has been associated with swallowing safety at Texture C. The study carried out by Ismael-Mohammed et al. in 2023 [27] concluded that 1500 ± 20% mPa·s (1200–1800 mPa·s) is an optimal and safe Texture C puree viscosity; therefore, this was the target value on which they were standardized. Second, the successful implementation of a quality control protocol to assess the rheological and textural parameters of the dishes. The daily results of this control were used by the kitchen team to adjust the properties of the TMD, which improved precision during elaboration.

These results highlight the importance of using objective measurements when evaluating the texture of TMD, as opposed to more subjective approaches such as those proposed by entities like IDDSI, BDA, or NDD. Quantitative analyses enable a far more precise, reproducible, and reliable assessment of textural properties, as they provide specific numerical values in International System units rather than relying on sensory or qualitative descriptors that are open to interpretation. This quantitative approach makes true standardization possible, defining measurable and comparable ranges between different hospital settings. In our study, standardization based on objective rheological parameters, particularly viscosity, was a key element in enabling consistent monitoring and control of quality. Therefore, adopting a quantitative, objective evaluation of texture should be considered essential to ensure the safety, homogeneity, and quality of diets provided to patients with dysphagia.

It is also worth noting that the effects of this standardization approach were more clearly defined for Texture C (thick puree) than for Texture E (fork-mashable). This difference can be explained by the intrinsic nature of each texture level. Purees are fluid systems, and their safety during swallowing depends primarily on viscosity, a parameter that is well characterized and for which clinically validated target ranges are available [28]. In contrast, Texture E involves soft solid foods that require active oral processing, including mastication, bolus formation, and fragmentation prior to swallowing. In this context, swallowing safety and efficacy depend on a complex interaction of multiple textural properties (such as hardness, cohesiveness, and adhesiveness) rather than on a single dominant parameter. The studies have not yet established quantitative reference values defining optimal or safe ranges for these properties in patients with OD, which limits the ability to define precise targets for standardization. As a consequence, although objective textural measurements were performed, the absence of validated reference thresholds limited the interpretation of Texture E data documentation and monitoring of textural variability, resulting in more heterogeneous and descriptive findings compared with pureed diets. Rather than representing a shortcoming of the intervention, these findings reflect current gaps in scientific knowledge and underscore the need for future studies aimed at identifying key quantitative parameters for fork-mashable solids. Establishing such reference values would be essential to achieve levels of standardization and quality control comparable to those successfully implemented for pureed textures.

The qualitative results indicate that patient-reported intake and acceptance were satisfactory in both 2023 and 2024. However, direct comparisons of mean scores between years should be interpreted with caution, as different assessment instruments were used (a 0–5 numerical scale in 2023 and a Face Likert Scale in 2024), which are not fully equivalent. Therefore, observed differences in scores may reflect characteristics of the questionnaires rather than true changes in patients’ perception. Overall, the results suggest favorable organoleptic and textural acceptance within each study year. This acceptance rate is comparable to findings from previous studies, which suggest that modifying the texture of the diet, when accompanied by an appropriate culinary approach, can improve adherence to treatment [15].

One limitation of TMD in hospital settings is that the nutritional requirements of patients are often increased, particularly in older adults with OD. The TMD model based on the triple adaptation of the diet explicitly addresses this issue by incorporating nutritional enrichment to ensure adequate energy and protein intake [17]. In patients with more severe clinical conditions, these requirements may be further elevated, making additional nutritional strategies necessary, such as oral nutritional supplements or the inclusion of protein-dense foods [30].

Recent studies have underscored the importance of biochemical and functional markers, such as oxidative stress parameters or adipokines, in the assessment of undernutrition among hospitalized older adults [31,32]. These markers reflect the systemic and functional consequences of inadequate nutritional intake and provide a broader perspective on geriatric malnutrition beyond dietary assessment alone. Although the present study did not evaluate such markers, our findings are complementary to this emerging evidence, as they address an upstream determinant of nutritional status by optimizing the safety, consistency, and acceptability of TMD.

The integration of these diets into the hospital circuit has enabled more efficient distribution and greater availability across the hospital. Follow-up qualitative evaluations further confirmed improvements in chewing perception, swallowing, and oral residue, reinforcing the idea that systematic and controlled texture modification can enhance the eating experience for patients.

All these results demonstrate that it is possible to standardize the rheological properties of TMD on a large-scale production level. The combination of quantitative and qualitative techniques enabled the design of a nutritionally complete diet. In addition, rheological and textural quality control ensured consistency with predefined texture standards considered appropriate for patients with OD. Furthermore, the high organoleptic quality has positively impacted the diet’s effectiveness, ensuring an intake rate of approximately 70%.

Looking forward, future perspectives include continuing to study the therapeutic effect of soft solid foods (BDA Texture E, IDDSI level 6) [22] to find the therapeutic range of textural parameters which guarantees similar levels of safety as that of purees. This will allow weekly monitoring to be expanded to include this texture, which will increase the quality of not only Texture C, but of the entire adapted diet in the hospital. Additionally, this large-scale standardization model should be replicated for fluids in the future. Nutrition and hydration are key elements that define a patient’s quality of life and health status; therefore, it is important to apply a similar effort to fluids. A large-scale hydration project is already being implemented for all patients with dysphagia in Mataró University Hospital, aiming to guarantee a minimum intake of 1.5 L of thick water at 250 mPa·s (optimal viscosity level that ensures high swallowing safety).

The present study has several limitations, as it is a primarily observational, single-center study that relies heavily on descriptive reporting, with limited statistical comparisons across years. Although the study demonstrates that the quality of TMD can be improved and safely monitored, the descriptive design limits the strength of causal inferences.

Secondly, the use of different assessment tools over the years made it difficult to compare palatability. The 2024 questionnaires were more specific and validated than those used in 2023, which may have contributed to observed differences and variability in the results. In addition, the sample size for the qualitative surveys was relatively small, particularly in 2024. This low response rate reflects the fact that surveys were part of routine clinical practice and no predefined number of respondents was established. Finally, it is also necessary to acknowledge the limitation arising from not having screened the surveyed patients by any criteria other than their ability to respond, which precluded the analysis of demographic and clinical characteristics such as age, sex, primary diagnosis, or functional and nutritional status. Taken together, differences in questionnaire structure, number of respondents and the lack of segregation by phenotypic profile introduce potential sampling bias and limit the reliability and comparability of patient satisfaction outcomes.

A third important limitation is that this study did not assess clinical variables, such as aspiration pneumonia, nutritional status, or length of hospital stay. While the literature suggests that our results are related to an improvement in patient health, this has not been specifically evaluated. This limits the strength of the clinical conclusions, and therefore, assessing the actual impact on patients may be an essential objective for future studies.

In the future, efforts should also focus on expanding this production and standardization protocol for TMD to other hospitals. In situations where objective and quantitative control of textured diets is not possible, a cold production line could be established to prepare and evaluate all diets in one location and then distribute them to multiple hospitals. Furthermore, more diverse TMD recipes should be developed, using ingredients other than thickeners, to allow for a wider range of possibilities in preparing these diets. This will not only increase the accessibility of these diets to the general population, but will also allow for adaptation to individual patient needs, such as intolerances, allergies and diet adaptations according to clinical requirements.

## 5. Conclusions

- The implementation of a weekly quality control of the rheological and textural properties of the TMD was associated with improved standardization of the viscosity of purees (Texture C, IDDSI level 4), maintaining values within a predefined range (1500 ± 20% mPa·s) considered appropriate for swallowing safety.
- The absence of well-established reference values for textural parameters related to swallowing safety in solid foods remains a limitation and may have constrained further improvements in the quality of Texture E (IDDSI level 6) TMDs.
- Patient-reported acceptance remained high during the study period, and the increased perception of ease of swallowing in 2024 suggests that the texture uniformity and consistency may play an important role in patient experience.
- Overall, the findings suggest that the combination of systematic OD screening, standardized TMD production, and ongoing quality control may represent a feasible framework for improving the nutritional and swallowing-related management of hospitalized patients with OD.
- Future research should include controlled and multicenter studies to: (1) identify optimal textural parameters that guarantee the safe swallowing of solids; (2) evaluate the impact of structured hydration protocols; (3) assess the transferability of large-scale standardization TMD production to cold production lines and to expand to other hospital settings; and (4) explore the development of more diverse TMDs that reduce reliance on thickening agents.

## Figures and Tables

**Table 1:** Prescription of texture-modified diets at Mataró Hospital in 2023. The values represent number of meals served per month and are reported descriptively. Text: Texture; IDDSI: International Dysphagia Diet Standardisation Initiative; Jan: January; Feb: February; Mar: March; Apr: April; Aug: August; Sep: September; Oct: October; Nov: November; Dec: December.

Text	Jan	Feb	Mar	Apr	May	June	July	Aug	Sep	Oct	Nov	Dec
Texture C (IDDSI 4)	320	354	339	432	688	606	428	609	576	590	586	727
Texture E (IDDSI 6)	87	161	241	284	398	378	351	327	349	249	379	307
TOTAL	407	515	580	716	1086	984	779	936	925	839	965	1034

**Table 2:** Prescription of texture-modified diets at Mataró Hospital in 2024. The values represent number of meals served per month and are reported descriptively. Text: Texture; IDDSI: International Dysphagia Diet Standardisation Initiative; Jan: January; Feb: February; Mar: March; Apr: April; Aug: August; Sep: September; Oct: October; Nov: November; Dec: December.

Text	Jan	Feb	Mar	Apr	May	June	July	Aug	Sep	Oct	Nov	Dec
Texture C (IDDSI 4)	1022	779	822	941	789	723	842	709	544	1002	858	873
Texture E (IDDSI 6)	407	436	418	444	400	324	304	354	330	376	616	493
TOTAL	1429	1215	1240	1385	1189	1003	1146	1063	874	1378	1474	1366

## Data Availability

The original contributions presented in this study are included in the article/Supplementary Material. Further inquiries can be directed to the corresponding author.

## Supplementary Materials

The following supporting information can be downloaded at: https://www.mdpi.com/article/10.3390/nu18040601/s1, Figure S1: Face Likert Scales. Table S1. Rheological and textural parameters of each studied TMD in Texture C in 2023. Table S2. Textural parameters of each studied TMD in Texture E in 2023. Table S3. Rheological and textural parameters of each studied TMD in Texture C in 2024. Table S4. Textural parameters of each studied TMD in Texture E in 2024.

## Abbreviations

The following abbreviations are used in this manuscript:

OD	Oropharyngeal Dysphagia
TMD	Texture-modified diets
WHO	World Health Organization
ICD	International Classification of Diseases
TP	Thickening products
AIMS-OD	Artificial Intelligence Massive Screening for Oropharyngeal Dysphagia
V-VST	Volume-Viscosity Swallowing Test
BDA	British Dietetic Association
JDD	Japanese Dysphagia Diet
NDD	National Dysphagia Diet
IDDSI	International Dysphagia Diet Standarisation Initiative
TPA	Texture Profile Analysis
PF	Peak force
AUC	Area under the curve
SD	Standard deviation

## Appendix A

**Table A1:** List of TMD selected for the study.

Texture Modified Diets	Designation	Ingredients	
		Texture C (Pureed)	Texture E (Fork-mashable)
Pollock fish	A	pollock fish (34.5%), salt (0.33%), olive oil (1.6%), black pepper (0.11%), thickener (0.22%), lemon sauce (62.3%)	pollock fish (62.1%), salt (0.6%), olive oil (2.6%), black pepper (0.2%), thickener (0.4%), lemon sauce (33.9%)
Cod fish	B	cod fish (53.0%), water (36.8%), olive oil (5.7%), thickener (1.0%)	cod fish (98.0%), thickener (2.0%)
Broccoli	C	broccoli (44.6%), ratatouille (8.9%), salt (0.2%), olive oil (1.8%), diced potato (24.7%), water (17.8%), thickener (2%)	broccoli (44.6%), ratatouille (8.9%), salt (0.2%), olive oil (1.8%), diced potato (24.7%), water (17.8%)
Pumpkin	D	pumpkin (80.4%), ratatouille (16.1%), salt (0.3%), olive oil (3.2%), thickener (2%)	diced pumpkin (80.4%), carrot (16.1%), salt (0.3%), 3.1% olive oil (3.1%), thickener (0.99%)
Cauliflower	E	cauliflower (44.6%), ratatouille (8.9%), salt (0.2%), olive oil (1.8%), diced potato (24.7%), water (17.8%), thickener (2%)	cauliflower (44.6%), ratatouille (8.9%), salt (0.2%), olive oil (1.8%), diced potato (24.7%), water (17.8%)
Turkey stew	F	sliced turkey breast (38.7%), salt (2.1%), olive oil (8.8%), black pepper (0.8%), thickener (1.1%), water (32.5%), concentrated chicken broth without salt (14.8%), vegetable sauce (32.5%), diced potato (22.3%), diced carrot (15.2%)	sliced turkey breast (23.1%), salt (1.3%), olive oil (5.3%), black pepper (0.5%), thickener (0.9%), water (18.4%), concentrated chicken, broth without salt (8.8%), ratatouille (19.4%), diced potato (13.3%), diced carrot (9.1%)
Mushroom stew	G	mushroom (51.3%), onion (10.6%), broth without salt (25.6%), water (9.2%), olive oil (2.5%)	mushroom (73.5%), onion (10.6%), broth without salt (12.5%), olive oil (1.3%)
Fideua	H	noodles nº2 (18.7%), olive oil (1.9%), salt (0.9%), ratatouille base (9.3%), stock (55%), peeled shrimp (5.6%), garlic and parsley (1.9%), mussels (5.6), thickener (1.1%)	noodles nº2 (18.7%), olive oil (1.9%), salt (0.9%), ratatouille base (9.3%), stock (55%), peeled shrimp (5.6%), garlic & parsley (1.9%), mussels (5.6), thickener (1.1%)
Lentil salad *	I	lentil (11.5%), ratatouille base (6.9%), potato (15.4%), carrot (19.2%), broth without salt (3.8%), water (38.4%), thickener (0.1%), olive oil (4.6%)	lentil (11.5%), ratatouille base (6.9%), potato (15.4%), carrot (19.2%), broth without salt (3.8%), water (15.4%), thickener (0.1%)
Red lentil	J	lentils (15.6%), ratatouille (31.3%), concentrated chicken broth (3.9%), salt (0.1%), olive oil (2%), diced potato (18.4%), diced carrot (7.8%), water (19.6%), thickener (1.2%), ground cumin (0.2%)	lentils (12.0%), ratatouille (7.2%), concentrated vegetable broth (4.0%), salt (0.7%), olive oil (2%), diced potato (16.0%), diced carrot (20.0%), water (40.0%), thickener (1.2%)
Hake fish	K	hake fish (94.2%), salt (0.9%), olive oil (3.8%), black paper (0.4%), thickener (0.7%)	hake fish (94.2%), salt (0.9%), olive oil (3.8%), black paper (0.4%), thickener (0.9%)
White pasta	L	pasta (19.4%), olive oil (1.9%), salt (1%), water (77.6%), thickener (0.1%)	pasta (97.0%), olive oil (1.9%), salt (1%), thickener (0.1%)
Soup pasta	M	pasta (19.4%), olive oil (1.9%), salt (1%), water (77.6%), thickener (0.1%)	pasta (97.0%), olive oil (1.9%), salt (1%), thickener (0.1%)
Potato	N	potato flakes (80%), olive oil (4.6%), thickener (0.3%), water (15.0%), salt (0.8%)	potato flakes (91.1%), olive oil (4.6%), thickener (0.3%), salt (0.8%)
Cooked turkey	O	turkey (51.6%), picada ajo (1.5%), water (43.0%), thickener (0.3%), olive oil (3.7%)	turkey (71.0%), picada ajo (1.5%), water (15%), thickener (0.1%), olive oil (3.7%)
Chicken	P	chicken burgermeat (43.5%), salt (0.4%), olive oil (1.4%), black pepper (0.1%), thickener (0.2%), water (36.2%), potato flakes (18.1%)	chicken burgermeat (63.5%), salt (0.4%), olive oil (1.4%), black pepper (0.1%), thickener (0.2%), water (10%), potato flakes (18.1%)
Veal	Q	veal (65.7%), potato (2.9%), olive oil (7.3%), water (23.4%), thickener (0.7%)	veal (84.0%), potato (2.9%), olive oil (7.3%), water (5.0%), thickener (0.3%)
Zucchini omelet	R	zucchini (44.6%), eggs (20.1%), diced potatoes (16.8%), salt (0.2%), virgin olive oil (1.8%), diced potatoes (26.7%), water (17.8%), thickener (1.2%)	zucchini (25.7%), eggs (30.8%), diced potatoes (41.1%), olive oil (1%), salt (0.9%), pepper (0.5%)
French omelet	S	french omelet (85%), water (11.1%), thickener (2.8%), olive oil (1.1%)	french omelet (70%), potato (25%), onion (2.5%), olive oil (2.5%)
Carrot	T	carrot (74.1%), water (18.5%), olive oil (7.4%), thickener (0.1%)	carrot (97.7%), olive oil (2.3%), thickener (0.1%)

* This dish is served cold.
