# Supplementary material for: Effect of a Large-Scale Production and Quality-Controlled Program for Texture-Modified Diets on Older Hospitalized Patients with Oropharyngeal Dysphagia
Source: Nutrients. 2026 Feb 11;18(4):601. doi: 10.3390/nu18040601 (PMC12943711; doi:10.3390/nu18040601)
Supplement: Supplementary file 1 [file nutrients-18-00601-s001.zip › nutrients-4030146-supplementary.pdf]

Effect of a large-scale production and quality-controlled program for texture modified diets on older hospitalized patients with oropharyngeal dysphagia

Supplementary material

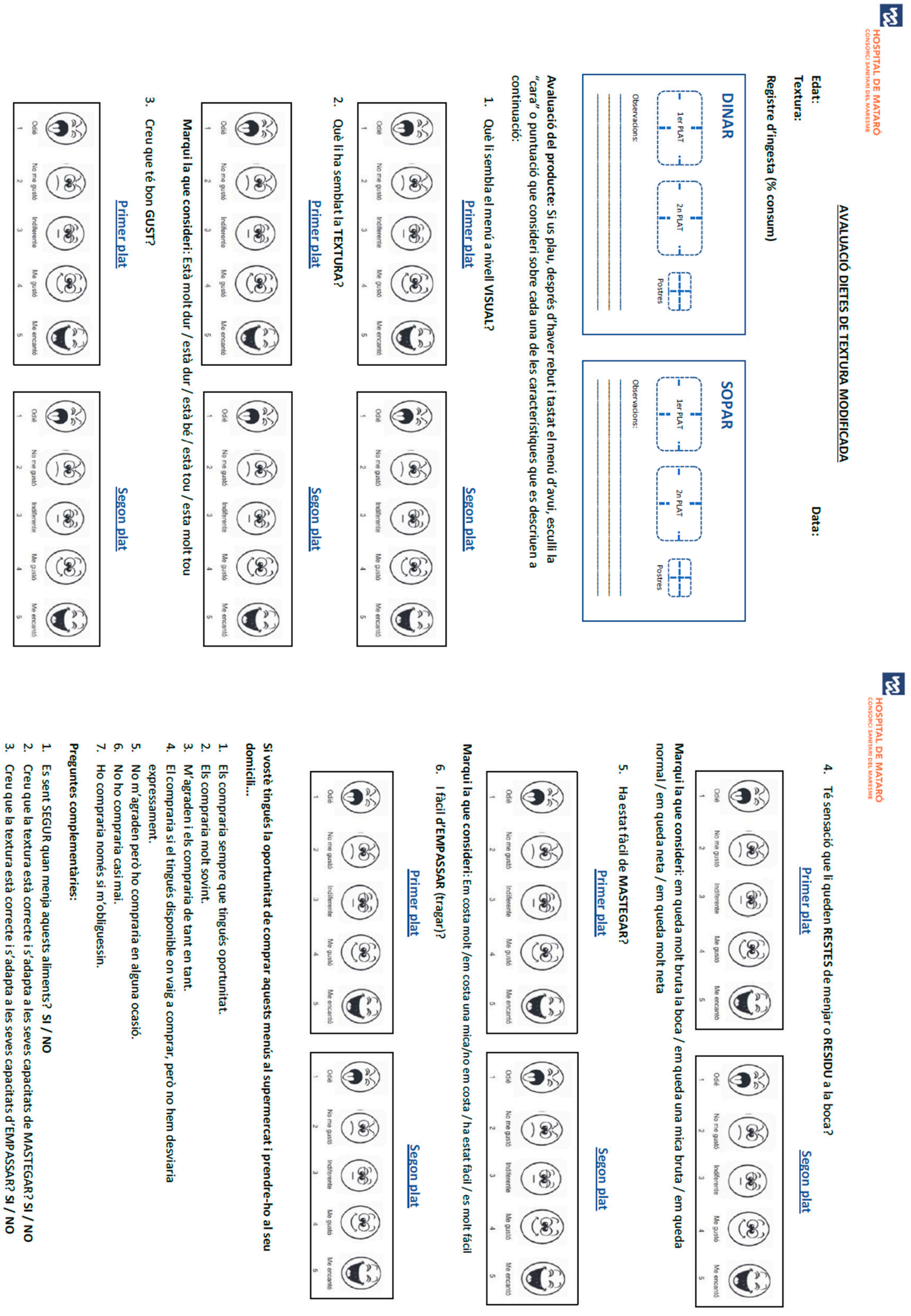

HOSPITAL DE MATARÓ
CONSORCI SANITARI DEL MARESME

AVALUACIÓ DIETES DE TEXTURA MODIFICADA

Edat: **Data:**
Textura:

Registre d'ingesta (% consum)

DINAR	SOPAR
1er PLAT / 2n PLAT / Postres	1er PLAT / 2n PLAT / Postres
Observacions:	Observacions:

Avaluació del producte: Si us plau, després d'haver rebut i tastat el menú d'avui, esculli la "cara" o puntuació que consideri sobre cada una de les característiques que es descriuen a continuació:

1. Què li sembla el menú a nivell **VISUAL?**

	Odié	No me gustó	Indiferente	Me gustó	Me encantó
Primer plat	1	2	3	4	5
Segon plat	1	2	3	4	5

2. Què li ha semblat la **TEXTURA?**

	Odié	No me gustó	Indiferente	Me gustó	Me encantó
Primer plat	1	2	3	4	5
Segon plat	1	2	3	4	5

Marqui la que consideri: Està molt dur / està dur / està bé / està tou / esta molt tou

3. Creu que té bon **GUST?**

	Odié	No me gustó	Indiferente	Me gustó	Me encantó
Primer plat	1	2	3	4	5
Segon plat	1	2	3	4	5

HOSPITAL DE MATARÓ
CONSORCI SANITARI DEL MARESME

4. Té sensació que li queden **RESTES** de menjar o **RESIDU** a la boca?

	Odié	No me gustó	Indiferente	Me gustó	Me encantó
Primer plat	1	2	3	4	5
Segon plat	1	2	3	4	5

Marqui la que consideri: em queda molt bruta la boca / em queda una mica bruta / em queda normal / em queda neta / em queda molt neta

5. Ha estat fàcil de **MASTEGAR?**

	Odié	No me gustó	Indiferente	Me gustó	Me encantó
Primer plat	1	2	3	4	5
Segon plat	1	2	3	4	5

Marqui la que consideri: Em costa molt /em costa una mica/no em costa / ha estat fàcil / es molt fàcil

6. I fàcil d'**EMPASSAR** (tragar)?

	Odié	No me gustó	Indiferente	Me gustó	Me encantó
Primer plat	1	2	3	4	5
Segon plat	1	2	3	4	5

Si vostè tingués la oportunitat de comprar aquests menús al supermercat i prendre-ho al seu domicili...

1. Els compraria sempre que tingués oportunitat.
2. Els compraria molt sovint.
3. M'agraden i els compraria de tant en tant.
4. El compraria si el tingués disponible on vaig a comprar, però no hem desviaria expressament.
5. No m'agraden però ho compraria en alguna ocasió.
6. No ho compraria casi mai.
7. Ho compraria només si m'obliguessin.

Preguntes complementàries:

1. Es sent SEGUR quan menja aquests aliments? **SI / NO**
2. Creu que la textura està correcte i s'adapta a les seves capacitats de MASTEGAR? **SI / NO**
3. Creu que la textura està correcte i s'adapta a les seves capacitats d'EMPASSAR? **SI / NO**

Figure S1: Face Likert Scales.

Table S1. Rheological and textural parameters of each studied TMD in Texture C in 2023.

TEXTURE C	Shear viscosity (mPa·s)	Maximum force (N)	Adhesiveness (N·s)	Cohesiveness
A	1527.06±350.64	0.57±0.08	0.81±0.18	0.78±0.05
B	1091.20±525.80	0.43±0.09	0.62±0.17	0.75±0.03
C	1095.34±171.63	0.53±0.11	0.90±0.23	0.82±0.04
D	1297.30±215.50	0.60±0.09	0.94±0.17	0.77±0.03
E	875.97±128.24	0.50±0.06	0.77±0.20	0.81±0.04
F	2093.76±414.48	0.61±0.11	0.89±0.03	0.76±0.05
G	670.43±148.82	0.37±0.02	0.52±0.06	0.78±0.02
H	4809.85±1618.21	0.73±0.31	0.94±0.53	0.73±0.15
I	1430.98±414.04	0.51±0.13	0.85±0.26	0.79±0.04
J	1551.56±379.99	0.54±0.08	0.90±0.14	0.80±0.03
K	1626.72±248.26	0.47±0.03	0.76±0.07	0.82±0.03
L	2188.65±396.06	0.60±0.14	0.96±0.28	0.80±0.04
M	4173.83±1525.77	0.95±0.19	1.26±0.26	0.75±0.08
N	2349.38±757.01	0.59±0.15	1.04±0.31	0.79±0.01
O	1317.35±543.80	0.57±0.25	0.75±0.29	0.73±0.07
P	1665.01±402.82	0.54±0.11	0.71±0.18	0.67±0.05
Q	1106.71±233.16	0.37±0.07	0.35±0.15	0.79±0.03
R	3111.48±1420.37	1.20±0.85	0.83±0.51	0.66±0.08
S	1996.01±600.54	0.63±0.11	0.93±0.20	0.73±0.03
T	863.84±176.91	0.41±0.03	0.57±0.11	0.79±0.03

Table S2. Textural parameters of each studied TMD in Texture E in 2023.

TEXTURE E	Maximum force (N)	Adhesiveness (N·s)	Cohesiveness
A	1.35±0.75	0.01±0.01	0.76±0.05
B	0.97±0.33	0.02±0.02	0.73±0.07
C	0.82±0.09	0.04±0.02	0.58±0.04
D	1.02±0.45	0.15±0.10	0.52±0.05
E	0.65±0.17	0.02±0.01	0.49±0.04
F	1.69±0.68	0.66±0.74	0.50±0.13
G	0.62±0.17	1.08±0.27	0.81±0.03
H	0.83±0.45	0.06±0.07	0.53±0.09
I	2.17±0.98	1.25±0.59	0.47±0.12
J	1.06±0.41	0.95±0.47	0.57±0.14
K	1.11±0.54	0.25±0.99	0.72±0.05
L	0.35±0.11	0.02±0.02	0.79±0.06
M	0.42±0.18	0.08±0.04	0.56±0.09
N	3.12±1.70	0.19±0.12	0.48±0.08
O	0.91±0.36	0.02±0.02	0.64±0.09
P	1.28±0.48	0.44±0.17	0.42±0.03
Q	2.02±0.60	0.93±0.50	0.51±0.09
R	2.73±1.40	0.04±0.02	0.66±0.05
S	1.06±0.81	0.12±0.45	0.87±0.03
T	1.01±0.39	0.03±0.04	0.61±0.07

Table S3. Rheological and textural parameters of each studied TMD in Texture C in 2024.

TEXTURE C	Shear viscosity (mPa·s)	Maximum force (N)	Adhesiveness (N·s)	Cohesiveness
A	1425.99±54.42	0.52±0.04	0.79±0.07	0.76±0.02
B	1365.44±57.21	0.52±0.03	0.72±0.04	0.78±0.03
C	1365.12±40.28	0.52±0.03	0.83±0.05	0.79±0.02
D	1299.99±35.47	0.52±0.02	0.79±0.05	0.77±0.02
E	1287.52±36.36	0.52±0.03	0.95±0.04	0.82±0.02
F	1142.45±86.02	0.42±0.01	0.53±0.03	0.74±0.02
G	1006.36±25.69	0.40±0.02	0.58±0.04	0.79±0.02
H	2047.79±105.41	0.44±0.02	0.65±0.04	0.82±0.02
I	1625.13±23.81	0.48±0.02	0.86±0.03	0.78±0.03
J	1831.50±110.07	0.60±0.04	0.98±0.11	0.81±0.03
K	1306.29±142.30	0.47±0.02	0.68±0.05	0.78±0.01
L	2415.91±128.68	0.46±0.03	0.75±0.07	0.84±0.02
M	2398.63±112.21	0.46±0.01	0.75±0.04	0.84±0.01
N	1689.05±101.60	0.61±0.04	0.92±0.06	0.75±0.03
O	1293.12±67.37	0.54±0.03	0.69±0.05	0.74±0.03
P	1702.62±58.80	0.73±0.05	1.02±0.07	0.70±0.01
Q	2175.15±146.33	0.53±0.02	0.70±0.06	0.74±0.01
R	2331.72±126.70	0.88±0.04	0.95±0.11	0.75±0.04
S	1962.71±53.71	0.51±0.02	0.71±0.04	0.75±0.02
T	1021.73±32.08	0.51±0.02	0.76±0.04	0.79±0.02

Table S4. Textural parameters of each studied TMD in Texture E in 2024.

TEXTURE E	Maximum force (N)	Adhesiveness (N·s)	Cohesiveness
A	1.37±0.46	0.04±0.07	0.69±0.07
B	1.17±0.39	0.02±0.02	0.73±0.04
C	0.71±0.14	0.07±0.02	0.52±0.02
D	0.82±0.23	0.51±0.26	0.50±0.08
E	0.88±0.11	0.16±0.04	0.57±0.03
F	1.60±0.71	0.40±0.33	0.45±0.04
G	0.93±0.26	1.12±0.25	0.63±0.06
H	0.71±0.24	0.07±0.11	0.51±0.12
I	1.03±0.29	1.39±0.45	0.72±0.06
J	1.04±0.29	0.92±0.25	0.57±0.06
K	1.02±0.39	0.04±0.03	0.67±0.07
L	0.36±0.08	0.22±0.13	0.76±0.07
M	0.55±0.18	0.06±0.03	0.59±0.06
N	1.35±0.50	0.12±0.14	0.35±0.08
O	1.71±0.49	0.06±0.03	0.52±0.03
P	1.75±0.61	0.66±0.36	0.46±0.06
Q	1.33±0.66	0.37±0.14	0.49±0.03
R	3.18±1.40	0.07±0.06	0.61±0.05
S	0.90±0.52	0.12±0.25	0.85±0.05
T	0.98±0.31	0.14±0.05	0.42±0.05
